# A blended preconception lifestyle programme for couples undergoing IVF: lessons learned from a multicentre randomized controlled trial

**DOI:** 10.1093/hropen/hoad036

**Published:** 2023-09-29

**Authors:** Tessy Boedt, Eline Dancet, Diane De Neubourg, Sofie Vereeck, Seghers Jan, Katleen Van der Gucht, Ben Van Calster, Carl Spiessens, Sharon Lie Fong, Christophe Matthys

**Affiliations:** Department of Chronic Diseases and Metabolism, KU Leuven, Leuven, Belgium; Department of Public Health and Primary Care, KU Leuven, Leuven, Belgium; Centre for Reproductive Medicine, University Hospitals Antwerp, Antwerp, Belgium; Centre for Reproductive Medicine, University Hospitals Antwerp, Antwerp, Belgium; Department of Movement Sciences, KU Leuven, Leuven, Belgium; Centre for Psychology of Learning and Experimental Psychopathology, KU Leuven, Leuven, Belgium; Department of Development and Regeneration, KU Leuven, Leuven, Belgium; Department of Development and Regeneration, KU Leuven, Leuven, Belgium; Leuven University Fertility Centre, University Hospitals Leuven, Leuven, Belgium; Department of Development and Regeneration, KU Leuven, Leuven, Belgium; Leuven University Fertility Centre, University Hospitals Leuven, Leuven, Belgium; Department of Chronic Diseases and Metabolism, KU Leuven, Leuven, Belgium; Department of Endocrinology, University Hospitals Leuven, Leuven, Belgium

**Keywords:** IVF, fertility, lifestyle, diet, physical activity, mindfulness, mobile health, randomized controlled trial

## Abstract

**STUDY QUESTION:**

What is the effect of a blended preconception lifestyle programme on reproductive and lifestyle outcomes of couples going through their first 12 months of IVF as compared to an attention control condition?

**SUMMARY ANSWER:**

This randomized controlled trial (RCT) was stopped prematurely because of the coronavirus disease 2019 (Covid-19) pandemic but the available data did not suggest that a blended preconception lifestyle programme could meaningfully affect time to ongoing pregnancy or other reproductive and lifestyle outcomes.

**WHAT IS KNOWN ALREADY:**

Increasing evidence shows associations between a healthy lifestyle and IVF success rates. Lifestyle programmes provided through a mobile phone application have yet to be evaluated by RCTs in couples undergoing IVF.

**STUDY DESIGN, SIZE, DURATION:**

A multicentre RCT (1:1) was carried out. The RCT started in January 2019 and was prematurely stopped because of the Covid-19 pandemic, leading to a reduced sample size (211 couples initiating IVF) and change in primary outcome (cumulative ongoing pregnancy to time to ongoing pregnancy).

**PARTICIPANTS/MATERIALS, SETTING, METHODS:**

Heterosexual couples initiating IVF in five fertility clinics were randomized between an attention control arm and an intervention arm for 12 months. The attention control arm received treatment information by mobile phone in addition to standard care. The intervention arm received the blended preconception lifestyle (PreLiFe)-programme in addition to standard care. The PreLiFe-programme included a mobile application, offering tailored advice and skills training on diet, physical activity and mindfulness, in combination with motivational interviewing over the telephone. The primary outcome was ‘time to ongoing pregnancy’. Secondary reproductive outcomes included the Core Outcome Measures for Infertility Trials and IVF discontinuation. Changes in the following secondary lifestyle outcomes over 3 and 6 months were studied in both partners: diet quality, fruit intake, vegetable intake, total moderate to vigorous physical activity, sedentary behaviour, emotional distress, quality of life, BMI, and waist circumference. Finally, in the intervention arm, acceptability of the programme was evaluated and actual use of the mobile application part of the programme was tracked. Analysis was according to intention to treat.

**MAIN RESULTS AND THE ROLE OF CHANCE:**

A total of 211 couples were randomized (105 control arm, 106 intervention arm). The hazard ratio of the intervention for time to ongoing pregnancy was 0.94 (95% CI 0.63 to 1.4). Little to no effect on other reproductive or lifestyle outcomes was identified. Although acceptability of the programme was good (6/10), considerable proportions of men (38%) and 9% of women did not actively use all the modules of the mobile application (diet, physical activity, or mindfulness).

**LIMITATIONS, REASONS FOR CAUTION:**

The findings of this RCT should be considered exploratory, as the Covid-19 pandemic limited its power and the actual use of the mobile application was low.

**WIDER IMPLICATIONS OF THE FINDINGS:**

This is the first multicentre RCT evaluating the effect of a blended preconception lifestyle programme for women and their partners undergoing IVF on both reproductive and lifestyle outcomes. This exploratory RCT highlights the need for further studies into optimal intervention characteristics and actual use of preconception lifestyle programmes, as well as RCTs evaluating effectiveness.

**STUDY FUNDING/COMPETING INTEREST(S):**

Supported by the Research foundation Flanders (Belgium) (FWO-TBM; reference: T005417N). No competing interests to declare.

**TRIAL REGISTRATION NUMBER:**

ClinicalTrials.gov Identifier: NCT03790449

**TRIAL REGISTRATION DATE:**

31 December 2018

**DATE OF FIRST PATIENT’S ENROLMENT:**

2 January 2019

WHAT DOES THIS MEAN FOR PATIENTS? This study looks at whether offering a blended lifestyle programme [combining a mobile phone application (app) with live contacts] to both partners of couples undergoing IVF makes a difference to their lifestyle and to their chances of IVF success. Several guidelines recommend addressing unhealthy lifestyle before trying to conceive in people with infertility, as increasing evidence shows that a healthy lifestyle increases the chances of IVF success. At present, a lifestyle programme is not routinely offered to couples undergoing IVF. Our group developed a blended lifestyle programme, including a mobile phone application with advice and skills training on diet, physical activity and mindfulness combined with motivational interviewing over the telephone. To assess the impact of this programme, couples beginning IVF were put into two groups: one group was given the mobile app treatment information in addition to standard care, while the other group also received the blended lifestyle programme. The results showed that lifestyle, time to ongoing pregnancy and other outcomes related to IVF success were not improved by the blended preconception lifestyle programme. Couples liked the blended lifestyle programme but actual use of the mobile app part of the programme, particularly among male partners, was low. Unfortunately, this study was stopped early because of the Covid-19 pandemic, so our results should be interpreted with caution. This trial highlights the need for further studies to optimize the content of a preconception lifestyle programme and establish how best to deliver this content to both partners undergoing IVF.

## Introduction

One in ten heterosexual couples faces infertility, defined as the failure to achieve a clinical pregnancy after at least 12 months of regular unprotected sexual intercourse (Boivin *et al.*, 2007; Mascarenhas *et al.*, 2012; Zegers-Hochschild *et al.*, 2017). About half of these couples seek fertility treatment, mainly involving IVF, with or without ICSI (Boivin *et al.*, 2007). Infertility and its treatment may impose a considerable emotional and financial burden on couples and society (Gameiro *et al.*, 2012; Wu *et al.*, 2013).

Several international guidelines recommend addressing an unhealthy lifestyle before attempting conception in people with infertility. ESHRE highlights the need for interdisciplinary developed programmes that meet the needs of infertile couples for advice on lifestyle behaviour and simultaneously meet their emotional, relational and cognitive needs. (Gameiro *et al.*, 2015). Similarly, the World Health Organization (WHO) recommends promoting a healthy lifestyle before conception (WHO, 2017, 2020). Addressing the factors that can be modified in order to improve the chance of having a healthy child is an important research priority for reproductive medicine (Moran *et al.*, 2016): lifestyle is one such factor. Increasing evidence shows that a healthy lifestyle is not only beneficial for people's general health but also for their reproductive health and the health of their children (Stephenson *et al.*, 2018). More specifically, a healthy diet, a normal BMI, and moderate physical activity are positively associated with IVF success rates (Vujkovic *et al.*, 2010; Rittenberg *et al.*, 2011; Twigt *et al.*, 2012; Salas-Huetos *et al.*, 2017; Sundaram *et al.*, 2017; Piché *et al.*, 2018; Rao *et al.*, 2018; van Dijk *et al.*, 2017). However, currently, IVF is not routinely combined with a lifestyle programme and there is a lack of guidance on the content and format regarding healthy lifestyle promotion for people with infertility (Homan *et al.*, 2018).

A recent Cochrane systematic review investigated the effect of preconception lifestyle advice for people with infertility, but did not provide clear guidance on the type of preconception advice that should be given or whether this advice helps to improve their chances of conceiving and their lifestyle (Boedt *et al.*, 2021b). This systematic review highlighted the need for high-quality randomized controlled trials (RCTs) investigating the effect of preconception lifestyle advice on relevant, well-defined clinical safety and effectiveness outcomes in both women and men with infertility (Duffy *et al.*, 2021).

To address the question of how to promote healthy lifestyle behaviour, recent evidence identified mobile health (mHealth) as a promising format for both the general population and couples with infertility (Afshin *et al.*, 2016; van Dijk *et al.*, 2016; van Dongen *et al.*, 2016). A Dutch group showed that a mHealth intervention targeting, amongst others, diet in people with infertility improved their lifestyle, especially when both partners participated (van Dijk *et al.*, 2016, 2017; Oostingh *et al.*, 2020). However, no differences in pregnancy rates were detected between the mHealth lifestyle intervention group (62.5%) and the control group (67.3%) and no other clinically relevant outcomes, including live birth, were reported (van Dijk *et al.*, 2016, 2017; Oostingh *et al.*, 2020). Based on thoroughly consulting the scientific evidence as well as experts and IVF patients, according to the steps specified by the Medical Research Council, our group developed a novel preconception blended lifestyle programme focusing on diet, physical activity and mindfulness, combining mHealth with consultation with a healthcare professional (Boedt *et al.*, 2021a).

Consequently, this RCT aimed to assess the effect of a novel systematically developed blended PREconception LiFestyle programme (PreLiFe-programme) offered to both partners going through their first year of IVF, on their reproductive and lifestyle outcomes.

## Materials and methods

### Study design

The PreLiFe-study was a multicentre, single-blind parallel RCT. CONSORT guidelines were followed and a detailed protocol of the study has been registered (ClinicalTrials.gov Identifier: NCT03790449) and published previously (Boedt *et al.*, 2019). Briefly, eligible couples starting IVF were randomized (1:1 allocation ratio) between an attention control arm or an intervention arm receiving the PreLiFe-programme for 12 months or until an ongoing pregnancy was confirmed by ultrasound at 12 weeks of gestational age. This study was approved by the Ethics Committee of the University Hospitals Leuven/KU Leuven and the local ethics committees of the participating clinics (approval number: s61596). The study started in January 2019 and was prematurely stopped on 13 March 2020 when, owing to the coronavirus disease 2019 (Covid-19) pandemic, all Belgian fertility clinics were closed for an undefined period. Recruitment was stopped and patients who had been randomized and still ongoing in the trial at that time needed to be censored. Supplementary Data File S1 provides an overview of the changes made to the predefined protocol because of the Covid-19 pandemic.

### Participants

Dutch speaking infertile heterosexual couples starting a first IVF/ICSI cycle, of whom women were ≤38 years of age and both partners had a smartphone, were enrolled at two university fertility clinics and three non-university fertility clinics located in Belgium. Couples previously treated with IVF/ICSI and/or who need preimplantation genetic testing or donor gametes were not eligible. In addition, couples were excluded if one of the partners had special dietary requirements or movement constraints, such as a broken leg, or other diseases of the musculoskeletal system.

### Randomization and blinding

After providing written informed consent, eligible couples were block randomized (stratified by clinic) by the researchers with an online password-protected programme to prevent disclosing the allocation sequence. Given the nature of the intervention, this was an open-label study where only the statistician was blinded.

### Interventions

All participating couples received standard medical treatment, i.e. IVF with or without ICSI, according to the local protocol of the participating fertility clinic. Both partners of couples randomized to the control arm received an attention control programme, which mimicked the amount of attention received by the intervention group but was thought not to have a specific effect (Aycock *et al.*, 2018). More specifically, the attention control arm received a mobile phone application (app) with medical treatment information detailing medication instructions and planned appointments. Both partners of couples randomized to the intervention arm additionally received the PreLiFe-programme. A detailed description of the PreLiFe-programme has been published previously (Boedt *et al.*, 2021a). In short, the PreLiFe-programme includes a mobile app with tailored advice and skills training on diet (food literacy), physical activity and mindfulness in combination with text messages and telephone interaction every 3 months with a health care professional, trained in motivational interviewing.

### Outcomes

Our predefined primary outcome was cumulative ongoing pregnancy rate within 12 months after randomization (COPR), with ongoing pregnancy defined as a viable intrauterine pregnancy of at least 12 weeks of gestational age confirmed by ultrasound. Based on advice from statisticians experienced in infertility trials, we changed to a time-to-event analysis for our primary outcome to be able to accommodate for censoring and to use the data of the many study patients who were in the midst of their 12 months study period. The updated primary outcome of this RCT was time to ongoing pregnancy after randomization.

Secondary outcomes covered reproductive outcomes and lifestyle outcomes. Secondary reproductive outcomes included the Core Outcome Measures for Infertility Trials (COMMIT), as it was recently recommended that these core outcome measures should be included in all infertility trials (Duffy *et al.*, 2021). The COMMIT includes: a viable intrauterine pregnancy confirmed by ultrasound (with a discernible heartbeat), pregnancy loss (miscarriage, ectopic pregnancy, abortion and stillbirth), live birth, gestational age at delivery, birthweight, neonatal mortality, and major congenital anomalies. In addition, differences in the occurrence of adverse events were collected. Finally, couples who did not return for treatment in the fertility clinic after failure of a previous cycle, were described as ‘IVF clinic discontinuation’. All reproductive outcomes were obtained from medical records.

Secondary lifestyle outcomes included: changes in diet quality (an index to reflect compliance with the food-based dietary guidelines) and fruit and vegetable intake in g/day (assessed with a food frequency questionnaire (Matthys *et al.*, 2015)); total moderate to vigorous physical activity and sedentary behaviour in minutes per week or day (assessed with the International Physical Activity Questionnaire (Craig *et al.*, 2003)); emotional distress (assessed with the Depression, Anxiety and Stress Scale Short Form (DASS-21) (de Beurs *et al.*, 2001)); BMI in kg/m^2^ (measured with scale and stadiometer); waist circumference in cm (measured with tape); and fertility-related quality of life (assessed with the Fertility Quality of Life Tool (Boivin *et al.*, 2011)). At baseline and every 3 months, these self-administered online lifestyle behaviour questionnaires were sent to participating couples through email and the mobile app. The follow-up measurements of BMI and waist circumference were planned about every 3 months, simultaneously with standard appointments during their IVF trajectory.

The acceptability of the PreLiFe-programme (assessed with the subjective quality scale of the Mobile App Rating Scale (Stoyanov *et al.*, 2015)) was evaluated in the intervention arm, at the end of the study. Actual use of the mobile app part of the PreLiFe-programme, defined as the percentage of participants having used all modules of the application at least one time, was objectively assessed with app-based tracking. Furthermore, we evaluated programme compliance (not withdrawing from the intervention) throughout the study. All data was captured in the Good Clinical Practice compliant Electronic Data Capture (EDC) platform, ‘Castor EDC’.

### Sample size

We determined the sample size for the original primary outcome (12-month ongoing pregnancy rate), assuming an intention-to-treat analysis (Boedt *et al.*, 2019). Couples who discontinued IVF treatment in participating clinics were considered negative for the primary outcome. Assuming a 50% versus 63% 12-month ongoing pregnancy rate in the control versus intervention arm, respectively, 230 couples per arm were required for a likelihood ratio chi-squared test with a power of 80% and a two-sided alpha of 5%. Calculations were performed using PASS14 software (PASS 14 Power Analysis and Sample Size Software (2015). NCSS, LLC. Kaysville, UT, USA, https://www.ncss.com/software/pass/).

### Statistical methods

Analysis was according to the intention-to-treat principle. Descriptive statistics were provided for baseline data, primary and secondary endpoints. The withdrawal rate was assessed and compared between the two arms.

For the updated primary outcome, namely time to ongoing pregnancy, the start of follow-up was the date of randomization. The end of follow-up was considered to be: date of confirmation of ongoing pregnancy (spontaneous and IVF pregnancies) at the 12 weeks ultrasound scan (Sunkara *et al.*, 2020); date on which the couple indicated to stop treatment (IVF clinic discontinuation); date on which the study prematurely stopped owing to Covid-19 (13 March 2020); or date on which a couple was 12 months in the study (study completion). Confirmation of ongoing pregnancy was the event of interest, whereas IVF clinic discontinuation was a competing event. Patients were censored after 12 months follow-up or on the 13 March 2020, whichever came first, if there was no confirmed ongoing pregnancy by that time. We generated cumulative incidence functions for the primary and competing events. Time to confirmation of ongoing pregnancy was analysed using the Fine and Gray subdistribution hazards regression, with study arm, women’s age and BMI at randomization as covariates. The subdistribution hazard ratio for the study arm was the key outcome, using an alpha of 5% to determine statistical significance.

For completeness, we also performed an exploratory analysis of the original primary outcome, namely cumulative ongoing pregnancy rate, within 12 months after randomization. This outcome could only be evaluated in the subset of couples randomized at least 1 year before the obligatory Covid-19 stop (13 March 2019 or earlier), so that 12 months of follow-up was possible (n = 51). The COPR in both groups was compared using logistic regression with the same covariates as the Fine and Gray model, and odds ratios (ORs) were reported.

Regarding the secondary reproductive outcomes, exploratory descriptive analyses were performed, but no effect estimates were reported.

Regarding the secondary lifestyle outcomes, the predefined protocol described the use of Mixed Models for Repeated Measurements. We decided to only include measures up to 6 months rather than 12 months, because of the low sample size following the Covid-19 stop. Supplementary Table S1 provides an overview of the number of patients still present in the study at each time point. The final analysis was based on mixed models with a random intercept for women and their partners separately (i.e. 18 models). Covariates were the baseline value, study arm, time (i.e. 3 months versus 6 months for questionnaire endpoints and actual time for BMI and waist circumference), the interaction between baseline and time, and the interaction between study arm and time. The joint *P*-value of the two coefficients involving study arm was obtained. The Kenward–Roger approach was used to calculate *P*-values.

Mixed models accommodate missing follow-up data under the missing at random approach (Molenberghs *et al.*, 2004). A minimal amount of missing values for baseline assessments of the secondary outcomes were observed and excluded. Secondary lifestyle data collected after confirmation of a pregnancy diagnosed by the detection of beta hCG in serum or urine was not included in the analyses. This implies that the results of the secondary analyses refer to measurements in the absence of pregnancy.

In the intervention arm, descriptive analyses were conducted on the programme compliance, acceptability and use of the PreLiFe-programme. All analyses were performed in R, using the packages of R, including rms, riskRegression, lme4, lmerTest (R Core Team (2022). R: A language and environment for statistical computing. R Foundation for Statistical Computing, Vienna, Austria). URL https://www.R-project.org/, https://CRAN.R-project.org/package=rms, https://CRAN.R-project.org/package=riskRegression, https://cran.r-project.org/web/packages/lme4/index.html. https://CRAN.R-project.org/package=lmerTest.

## Results

**Figure 1.**
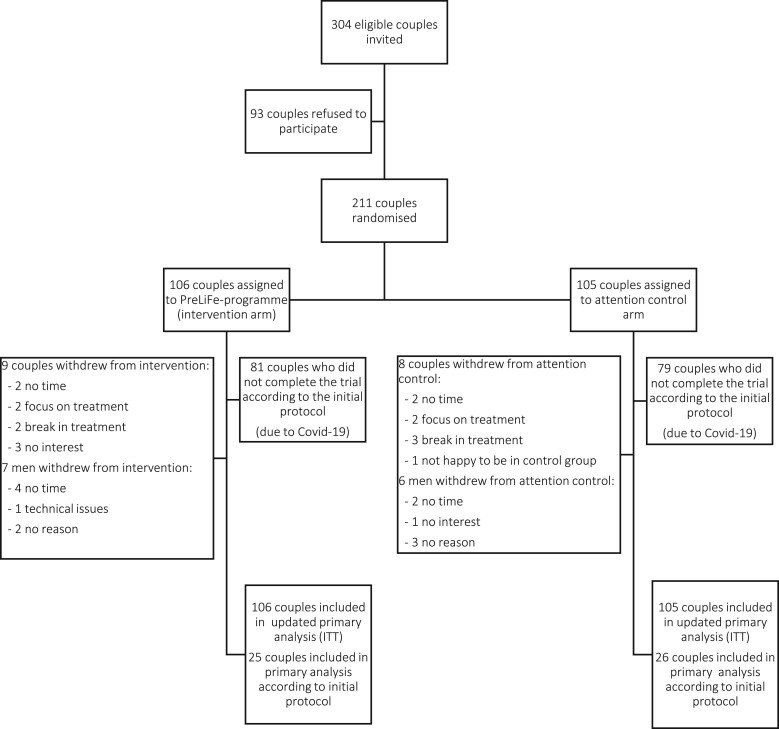
**Patient recruitment, randomization, and follow-up in a randomized controlled trial of improvements in lifestyle and IVF outcome.** A novel programme designed to improve lifestyle was offered to both partners going through their first year of IVF, and impact on reproductive and lifestyle outcomes was assessed. The primary outcome was time to ongoing pregnancy. Couples were randomized between an attention control arm and an intervention arm receiving a blended lifestyle programme for 12 months in addition to standard care. PreLiFe-programme, PREconception LiFestyle programme; ITT, intention to treat; Covid-19, coronavirus disease 2019.

Figure 1, the CONSORT flowchart, provides an overview of patient recruitment, randomization and follow-up. Between 2 January 2019 and 13 March 2020, we identified 304 eligible couples. Among these, 211 couples (69%) provided informed consent and were randomized to the attention control arm (n = 105) or intervention arm (n = 106). Overall, a total of 17 (8%) couples and 13 (6%) additional men withdrew from the study. The withdrawal rate was low and balanced between groups. Reasons for study withdrawal are presented in Fig. 1. In addition, Supplementary Table S1 provides an overview of the number of couples in the study at each time point and their reasons for having ended the study. Baseline characteristics of participants according to study arm are presented in Table 1. Approximately 70% of the couples were highly educated and more than 90% of the couples had a European ethnicity. Average (±SD) BMI was 24.6 (±4.3) kg/m^2^ in women and 26 (±4.0) kg/m^2^ in men. The mean duration of time trying to conceive was circa 2 years and one in ten couples had unexplained infertility.

**Table 1:** Baseline characteristics for couples in a randomized controlled trial, according to study arm.

	Women			Men	
Characteristic	**Control** (n = 105)	**Intervention** (n = 106)		**Control** (n = 105)	**Intervention** (n = 106)
Age, mean (SD), years	30.5 (3.5)	30.7 (3.9)		34.2 (5.5)	33.9 (6.1)
BMI, mean, (SD), kg/m²^a^					
Underweight (BMI < 18.5 kg/m²)	3 (2.9%)	2 (1.9%)		1 (1%)	2 (1.9%)
Normal weight (BMI = 18.5–24.99 kg/m²)	67 (64%)	58 (55%)		38 (37%)	38 (36%)
Overweight (BMI = 25–29.99 kg/m²)	21 (20%)	30 (28%)		50 (48%)	52 (50%)
Obesity (BMI ≥ 30 kg/m²)	14 (13%)	16 (15%)		15 (14%)	13 (12%)
Unknown	0	0		1	1
No. (%) European ethnicity^b^	99 (94%)	99 (93%)		103 (98%)	101 (95%)
Education^b^					
n (%) ISCED levels 0–3	19 (18%)	20 (19%)		33 (31%)	34 (32%)
n (%) ISCED levels 4–6	52 (50%)	59 (56%)		38 (36%)	48 (45%)
n (%) ISCED levels 7–8	34 (32%)	27 (25%)		34 (32%)	24 (23%)
n (%) Smoking^b^	14 (13%)	7 (7%)		22 (21%)	19 (18%)
n (%) Alcohol intake^b^	19 (18%)	29 (27%)		11 (10%)	13 (12%)
Unknown	2	1		12	8
n (%) Medical condition^b^	22 (21%)	23 (22%)		20 (19%)	24 (23%)
n (%) Medication use past 3 months^b^	25 (24%)	24 (23%)		20 (19%)	28 (26%)
n (%) Folic acid supplement use^b^	70 (67%)	63 (59%)		NA	NA
n (%) Following complementary therapy^b^	12 (11%)	18 (17%)		4 (3.8%)	2 (1.9%)
Duration of time trying to conceive, mean (SD), months^b^	26 (15)	27 (16)	Similar for male partner (couple outcome)		
Type of infertility^c^					
n (%) Primary	75 (71%)	79 (75%)		73 (70%)	78 (74%)
n (%) Secondary	30 (29%)	27 (25%)		32 (30%)	28 (26%)
Female factor infertility diagnosis^c^			Male factor infertility diagnosis^c^		
n (%) No	41 (39%)	52 (49%)	n (%) No	35 (33%)	33 (31%)
n (%) Yes	52 (50%)	52 (49%)	n (%) Yes	58 (55%)	71 (67%)
n (%) Unexplained	12 (11%)	2 (2%)	n (%) Unexplained	12 (11%)	2 (2%)

n = number of participants.

a Measured and calculated as weight in kilograms, divided by height in meters squared.

b Self-reported.

c Infertility diagnosis at baseline was obtained from medical files.

A novel programme designed to improve lifestyle was offered to both partners going through their first year of IVF, and impact on reproductive and lifestyle outcomes was assessed. The control arm received treatment information by mobile phone in addition to standard care. The intervention arm received the blended preconception lifestyle (PreLiFe)-programme in addition to standard care.

### Primary outcome

**Figure 2.**
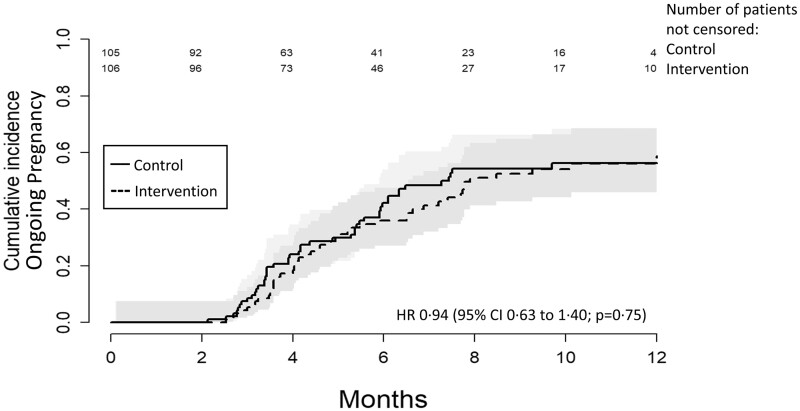
**Cumulative incidence function of ongoing pregnancy over time by study arm.** HR, hazard ratio.

An overview on the cumulative incidence of ongoing pregnancy over time by study arm is presented in Fig. 2. The sub-distribution hazard ratio of ongoing pregnancy for the intervention versus attention control arm was 0.94 (95% CI 0.63 to 1.40, *P* = 0.75). Among couples with confirmed ongoing pregnancy, the mean time to ongoing pregnancy was 4.79 (±2.38) months in the intervention arm and 4.11 (±1.68) months in the attention control arm.

Supplementary Table S2 provides an overview of our originally planned primary analysis: cumulative ongoing pregnancy within 12 months after randomization in the subset of couples who completed our trial according to the initial protocol (n = 51). The OR of cumulative ongoing pregnancy within 12 months for couples in the intervention versus attention control arm was 0.93 (95% CI 0.27 to 3.20).

### Secondary reproductive outcomes

A descriptive overview of the other reproductive outcomes that occurred during the study period of participating couples is presented in Table 2. IVF clinic discontinuation occurred 12 times in each group. Miscarriage occurred seven times in each group. There was one adverse event in the intervention arm (i.e. a torsion of the left ovary). Live birth occurred in 45 (42%) couples of the intervention group and 41 (39%) couples of the attention control group. One couple in the intervention group conceived twins. No neonatal mortality or major congenital anomalies were observed.

**Table 2:** Descriptive overview of reproductive outcomes in the randomized controlled trial, according to study arm.

Outcome	**Control** (n = 105)		**Intervention** (n = 106)	
n (%) IVF clinic discontinuation (total)	12	(11%)	12	(11%)
n (%) Doctor initiated clinic discontinuation	1	(1%)	1	(1%)
n (%) Patient initiated clinic discontinuation: 2nd opinion	7	(7%)	7	(7%)
n (%) Patient initiated clinic discontinuation: psychosocial reasons	5	(5%)	3	(3%)
n (%) Clinical pregnancy (evidence of intrauterine gestational sac, confirmed by US)	52	(50%)	52	(49%)
n (%) Clinical pregnancy with fetal heartbeat (viable intrauterine pregnancy confirmed by US)	49	(47%)	50	(47%)
n (%) Ongoing pregnancy (viable intrauterine pregnancy confirmed by US of at least 12 weeks	47	(45%)	48	(45%)
n (%) Ongoing IVF pregnancy	44	(42%)	44^a^	(42%)
n (%) Ongoing spontaneous pregnancy	3	(3%)	3	(3%)
n (%) Ongoing IUI pregnancy	0	(0%)	1	(1%)
Time to ongoing pregnancy, mean (SD), months	4.11	(1.68)	4.79	(2.38)
n (%) Adverse events	0	(0%)	1^b^	(1%)
Pregnancy loss				
n (%) Miscarriage	7^c^	(7%)	7^c^	(7%)
n (%) Ectopic pregnancy	1	(1%)	1	(1%)
n (%) Abortion	0	(0%)	2^d^	(2%)
n (%) Still birth	0	(0%)	0	(0%)
n (%) Multiple gestation (twins)	0	(0%)	1	(1%)
n (%) Live birth	41	(39%)	45^a^	(42%)
Birthweight, mean (SD), grams	3379	(546)	3255	(438)
Gestational age at delivery, mean (SD), weeks of gestation	39	(1.56)	38.6	(1.34)
n (%) Neonatal mortality	0/41	(0%)	0/45	(0%)
n (%) Major congenital anomalies	0/41	(0%)	0/45	(0%)

n = number of participants.

a Twins counted as one event.

b Torsion left ovary.

c Two times in one person in intervention and control.

d Two times in same couple (heart condition).

The control arm received treatment information by mobile phone in addition to standard care. The intervention arm received the blended preconception lifestyle (PreLiFe)-programme in addition to standard care.

### Secondary lifestyle-related outcomes

An overview of the descriptive statistics for all lifestyle outcomes over time and by study arm, including the joint *P*-values of the mixed model analyses, is presented (Table 3, Supplementary Fig. S1). Details of the mixed model analyses are presented in Supplementary Table S3. Results for the lifestyle-related outcomes at 6 months were available for 34 women and 26 partners. Our results did not suggest clear effects of the PreLiFe-programme on these lifestyle-related outcomes, although uncertainty is substantial owing to the small sample size. For women, there was a statistically significant result for dietary outcomes, regarding the intervention.

**Table 3:** Overview of the descriptive statistics for all lifestyle-related outcomes over time and by study arm, including the joint *P*-values of the mixed model analyses.

Women					Men				Joint *P*-values for arm and time arm
	Control		Intervention		Control		Intervention		
Months	n^a^	Mean (SE)	n^a^	Mean (SE)	n^a^	Mean (SE)	n^a^	Mean (SE)	
**Diet quality (%)**									♀ = 0.016 ♂ = 0.135
0	97/105 (92%)	72.1 (1.1)	99/106 (93%)	72.1 (1.3)	84/105 (80%)	67.5 (1.5)	89/106 (84%)	66.6 (1.4)	
3	40/63 (63%)	74.3 (1.5)	47/66 (71%)	76.6 (1.4)	28/63 (44%)	68.4 (2.4)	35/66 (53%)	69 (2.1)	
6	13/32 (41%)	77.3 (2.4)	21/36 (58%)	76.3 (1.8)	10/32 (31%)	61.9 (3.7)	16/36 (44%)	74.5 (2.2)	
**Fruit intake (g/day)**									♀ = 0.017 ♂ = 0.108
0	97/105 (92%)	141.3 (9.5)	99/106 (93%)	123.7 (9.7)	84/105 (80%)	107.5 (10.1)	89/106 (84%)	112.8 (12.5)	
3	40/63 (63%)	146.9 (14.9)	47/66 (71%)	144.3 (12.9)	28/63 (44%)	113.6 (16.6)	35/66 (53%)	107.8 (13.8)	
6	13/32 (41%)	203.2 (20.9)	21/36 (58%)	129.5 (17.9)	10/32 (31%)	59.4 (17.4)	16/36 (44%)	177.5 (31)	
**Vegetable intake (g/day)**									♀ = 0.011 ♂ = 0.126
0	97/105 (92%)	152.9 (8.2)	99/106 (93%)	150.1 (9.3)	84/105 (80%)	155.2 (10.4)	89/106 (84%)	123.1 (8.5)	
3	40/63 (63%)	133 (11.9)	47/66 (71%)	188.2 (13.8)	28/63 (44%)	129 (19.8)	35/66 (53%)	144 (11.4)	
6	13/32 (41%)	139.1 (23)	21/36 (58%)	162.4 (22.4)	10/32 (31%)	134.9 (27.1)	16/36 (44%)	142.5 (16.6)	
**Total moderate to vigorous physical activity (minutes/week)**									♀ = 0.777 ♂ = 0.463
0	97/105 (92%)	658.6 (60.9)	99/106 (93%)	686.2 (72.4)	84/105 (80%)	855.2 (72.8)	89/106 (84%)	807.8 (76.9)	
3	40/63 (63%)	548.6 (74.1)	47/66 (71%)	605.4 (103)	28/63 (44%)	848.2 (119.9)	35/66 (53%)	597 (88.9)	
6	13/32 (41%)	448.8 (141.6)	21/36 (58%)	481.4 (103.2)	10/32 (31%)	625.5 (143.6)	16/36 (44%)	623.4 (137.5)	
**Sedentary behaviour (min/day)**									♀ = 0.673 ♂ = 0.559
0	97/105 (92%)	371.9 (21.9)	99/106 (93%)	374.8 (23.4)	84/105 (80%)	357.3 (24.9)	89/106 (84%)	391.3 (23.1)	
3	40/63 (63%)	349.1 (36.2)	47/66 (71%)	360 (36.8)	28/63 (44%)	333.8 (38)	35/66 (53%)	402 (33.9)	
6	13/32 (41%)	409.6 (49.4)	21/36 (58%)	305 (31.6)	10/32 (31%)	351 (53.3)	16/36 (44%)	381.6 (56.2)	
**Emotional distress (total DASS-21 score)**									♀ = 0.554 ♂ = 0.528
0	97/105 (92%)	26.5 (1.7)	99/106 (93%)	29.2 (2)	84/105 (80%)	18.8 (1.7)	89/106 (84%)	20.9 (1.5)	
3	40/63 (63%)	24.4 (3.1)	47/66 (71%)	26.2 (2.8)	28/63 (44%)	16.2 (2.5)	35/66 (53%)	16.5 (2.1)	
6	13/32 (41%)	31.2 (6)	21/36 (58%)	28.3 (4.5)	10/32 (31%)	11.4 (5.2)	16/36 (44%)	19.1 (3.9)	
**Fertility related quality of life (total FERTIQOL score)**									♀ = 0.697 ♂ = 0.111
0	97/105 (92%)	71.9 (1.2)	99/106 (93%)	70.9 (1.2)	84/105 (80%)	80.4 (1.1)	89/106 (84%)	79.2 (1.2)	
3	40/63 (63%)	69.7 (2)	47/66 (71%)	68.2 (1.8)	28/63 (44%)	77.8 (1.8)	35/66 (53%)	80.2 (1.6)	
6	13/32 (41%)	68.5 (3.7)	21/36 (58%)	65.6 (2.8)	10/32 (31%)	82.9 (2.8)	16/36 (44%)	75.8 (3.4)	
BMI (kg/m²)^b^									♀ = 0.329 ♂ = 0.429
Waist circumference (cm)^b^									♀ = 0.750 ♂ = 0.032

a Number of patients with data available (nominator), number of patients still in the study at that time.

b See Supplementary Fig. S1 for descriptive representation of BMI and waist circumference. DASS, Depression, Anxiety and Stress Scale; n, number of participants.

The control arm received treatment information by mobile phone in addition to standard care. The intervention arm received the blended preconception lifestyle (PreLiFe)-programme in addition to standard care.

### Compliance, acceptability and actual use

Compliance to the programme was 91.5%. Reasons for withdrawal are presented in Fig. 1.

Acceptability of the programme revealed an average score of 6/10 (±1 SD) for women and men (range 3 to 8). However, actual use of the mobile app part of the PreLiFe-programme was limited (Supplementary Table S4). Fifteen (14%) couples used every component of the app. Forty (38%) men and 10 (9%) women did not actively use any app module, and two men and two women did not initiate the app.

## Discussion

To our knowledge, this is the first multicentre RCT evaluating the effect of a blended preconception lifestyle programme on relevant reproductive and lifestyle outcomes in both partners going through IVF. Our results indicate little or no effect of this PreLiFe-programme on relevant reproductive outcomes, as defined by COMMIT and on the lifestyle of both partners. However, the compromised sample size owing to the Covid-19 pandemic causing a premature stop to our RCT, the large CIs and the limited actual use of the mobile app part of our programme resulted in an inability to draw firm conclusions on the potential effect of preconception lifestyle programmes for couples undergoing IVF. Therefore, this study should be considered exploratory. The results of this study may provide future large scale RCTs with insights into the effect sizes to expect. Furthermore, the insights into objective use of the mobile app part of our programme provides an opportunity for future studies to further determine optimal intervention characteristics of preconception lifestyle programmes.

Despite our effort to meticulously develop a preconception lifestyle programme based on the needs of IVF patients, expert opinion and scientific evidence, only half of the women and one in five men used all modules of the mobile app part of our programme. Comparison of the use of (preconception) lifestyle interventions across studies is difficult given the large variability in intervention characteristics across studies and in the reporting of use across studies (Milne-Ives *et al.*, 2020). A recent Dutch study providing tailored coaching on vegetable, fruit, and folic acid supplement intake, as well as smoking and alcohol consumption through text messages or e-mail to couples trying to conceive, showed an overall programme compliance (not withdrawing from the intervention) of 73.6% (Oostingh *et al.*, 2020). Although they did not report on the actual use of the intervention, e.g. reading the messages and/or e-mail, lifestyle behaviour improved after the intervention as compared to control. Nevertheless, no differences in pregnancy rates between the intervention (62.5%) and control arm (67.3%) were observed (van Dijk *et al.*, 2016, 2017; Oostingh *et al.*, 2020). As shown in our study, programme compliance (in terms of not withdrawing from the intervention group) does not reflect the actual use of the programme (components). Our study showed high compliance to the intervention group (91.5%) but use of the mobile app part of our programme was lower, especially in male partners. Although our previous research showed that patients undergoing IVF need support in lifestyle modification and prefer a mobile app as a time-efficient intervention format (Boedt *et al*., 2021a) the actual use of such an application was disappointing. A possible reason may be found in a recent study by Robertson *et al.* (2022). Patients undergoing IVF might prefer focusing on their treatment instead of other factors, such as emotional management or their lifestyle, leading to reduced chances of achieving lifestyle behaviour change (Robertson *et al.*, 2022). Indeed, our PreLiFe-programme was offered immediately before the start of IVF without a fixed time free from IVF and this may have given some couples too little time to follow and use all the modules of the PreLiFe-programme and implement lifestyle changes during their first IVF cycle. In addition, only couples starting a first IVF attempt were included. It may be hypothesized that these patients prefer to focus more on the medical treatment, rather than on lifestyle. Future studies should further determine the optimal characteristics of the intervention.

This RCT had some possible limitations, not related to the sample size and limited use. Self-selection of the participants might have played a role. As compared to the overall Belgian population, our study population had a higher physical activity level and lower rates of overweight and obesity (Gisle, 2014; WHO, 2022). Participating in our study could be associated with an interest in a healthy lifestyle, leaving less room for improvement in lifestyle behaviour. The RCT was single-blinded as the nature of the intervention only allowed blinding the statistician. Bias was unlikely in the assessment of the reproductive outcomes, but the lifestyle outcomes assessed by validated questionnaires were susceptible to socially desirable answers and recall bias. Another limitation of this study was that most patients were Caucasian and highly educated, limiting the generalizability of our results.

This RCT had several strengths. First, we followed a systematic and thorough intervention development process with a solid theoretical base and took account of the needs of patients, expert opinion and scientific evidence (Craig *et al.*, 2013). We reported in detail our intervention characteristics (Boedt *et al.*, 2021a) and the protocol for intervention assessment (Boedt *et al.*, 2019) to limit the risk of bias and enable future researchers to identify optimal intervention characteristics of preconception lifestyle interventions. Future multiple arm comparative trials are warranted to assess different intervention characteristics including behaviour change strategies, lifestyle components, and intervention channels. Second, to our knowledge, we are the first to measure the actual use of the mobile app part of a preconception lifestyle programme. We did so using app-based tracking, an objective measure not prone to social desirability. Further analysis of use data will lead to a better understanding of (dis)use of such mobile apps and this can contribute to identifying and improving factors that lead to higher use and engagement, ultimately enhancing the effectiveness of (mobile) preconception lifestyle interventions (Perski *et al.*, 2016; Kelders *et al.*, 2020). Third, we addressed both partners of IVF couples, rather than only the woman. Involving both partners in lifestyle interventions may facilitate mutual support, behaviour change and programme compliance at little additional cost (Best *et al.*, 2017; van Dijk *et al.*, 2017). Finally, we assessed the core outcome measures for infertility trials (Duffy *et al.*, 2021). This will enable future research to better compare, combine, and synthesize data to generate solid evidence to inform clinical practice.

In conclusion, this exploratory study did not provide solid evidence on the effect of a preconception lifestyle programme in couples undergoing IVF. Given the needs of both patients and health care professionals and the potential health benefits, further exploration of optimal intervention characteristics of preconception lifestyle programmes should remain a topic of interest for future research. Future studies should also try to identify factors to improve engagement with preconception lifestyle programmes to ultimately identify the most effective means of encouraging a healthy lifestyle in people undergoing IVF.

## Supplementary Material

hoad036_Supplementary_Data

## Data Availability

De-identified participant data from this RCT will be shared on reasonable request to the corresponding author.
