# Supplementary material for: A blended preconception lifestyle programme for couples undergoing IVF: lessons learned from a multicentre randomized controlled trial
Source: Hum Reprod Open. 2023 Sep 29;2023(4):hoad036. doi: 10.1093/hropen/hoad036 (PMC10918763; doi:10.1093/hropen/hoad036)
Supplement: hoad036_Supplementary_Data [file hoad036_supplementary_data.docx]

**Supplementary Material**

**Supplementary Data File S1.** Overview of the changes made to the predefined protocol due to the Covid-19 pandemic.

The Covid-19 pandemic put a hold on all IVF trajectories in Belgian fertility clinics for a undefined period, therefore this RCT prematurely stopped in March 2020. At that point, 211 of 460 intended couples were randomised and 51 couples completed the trial as planned. Eighty patients who had been randomised but were still enrolled in the trial needed to be censored. Consequently, we had to make some adaptions to the predefined protocol. In collaboration with a statistical team, the following modifications were established (added in orange to the original overview figure of our RCT as published in BMJ Open (1)):


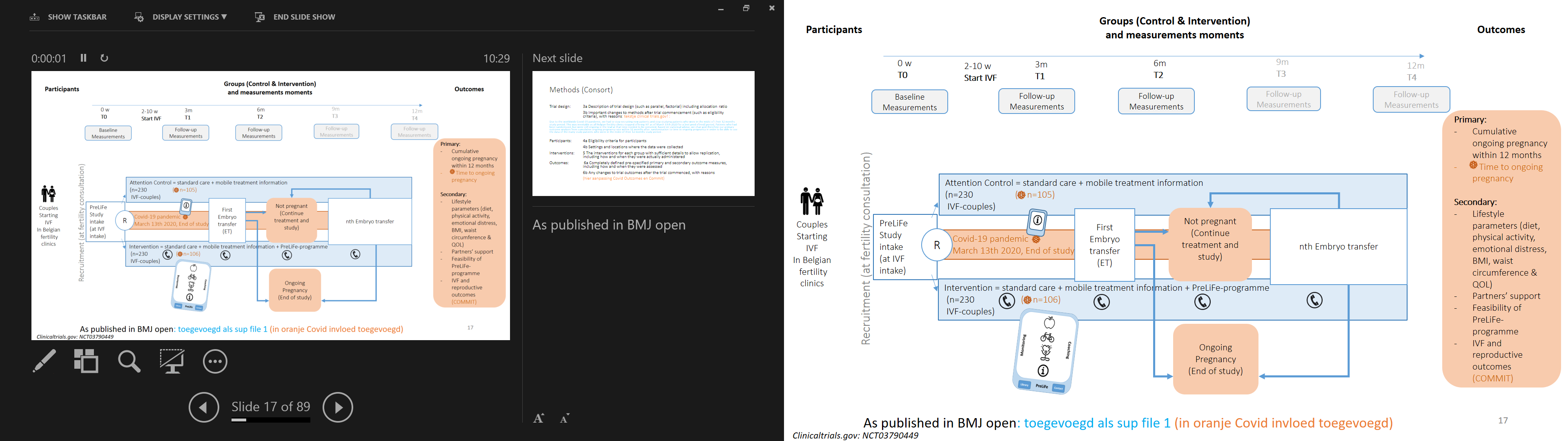


***Overview of the PreLiFe-RCT (with in orange changes made due to the Covid-19 stop).*** *BMI = Body Mass Index, IVF = In Vitro Fertilisation, QOL = Quality of LiFe, RCT = Randomised Controlled Trial, COMMIT = Core Outcome Measures for Infertility Trials*

- The primary outcome analysis changed from cumulative ongoing pregnancy rate (COPR) within 12 months after randomisation to time-to-event (time to ongoing pregnancy) analysis to be able to accommodate for censoring and to use the data of the many study patients who were in the midst of their 12 months study period. The original primary outcome, COPR, could only be evaluated in the subset of couples who were randomised at least one year before the obligatory Covid-19 stop (13th of March 2019 or earlier), such that 12 months of follow-up was possible (n=51).
- The secondary lifestyle outcomes were evaluated over three and six months after randomisation, rather than three, six, nine and twelve months. Limited questionnaire results were available at these later months due to the Covid-19 stop (see supplementary File 2). Instead of using mixed models for repeated measurements (MMRM) the analysis was based on mixed models with a random intercept.
- During this project, Duffy *et al.* identified a core outcome set for infertility trials (COMMIT) to help standardise outcome selection, collection, and reporting (2). This infertility core outcome set should be included in all infertility trials and was therefore additionally incorporated into our study.

1. *Boedt T, Dancet E, Lie Fong S, Peeraer K, De Neubourg D, Pelckmans S et al. Effectiveness of a mobile preconception lifestyle programme in couples undergoing in vitro fertilization (IVF): the protocol for the PreLiFe randomised controlled trial (PreLiFe-RCT). BMJ OPEN. 2019; 9 (7), Art.No. ARTN e029665.*
2. *Duffy JMN, Bhattacharya S, Bhattacharya S, Bofill M, Collura B, Curtis C, et al. Standardizing definitions and reporting guidelines for the infertility core outcome set: an international consensus development study. Fertil Steril. 2021;115(1):201-12.*

**Supplementary Table S1.** Patient follow-up per study.

| **Months** | **Arm** | **In study**  **(n)** | **Out owing to pregnancy (still pregnancy follow-up until 12 weeks)**  **(n)** | **Out owing to IVF clinic discontinuation**  **(n)** | **Out owing to Covid-19 stop**  **(n)** |
| --- | --- | --- | --- | --- | --- |
| 0 | Control | 105 | 0 | 0 | 0 |
| 0 | Intervention | 106 | 0 | 0 | 0 |
| 3 | Control | 63 | 26 | 2 | 14 |
| 3 | Intervention | 66 | 26 | 1 | 13 |
| 6 | Control | 32 | 20 | 4 | 7 |
| 6 | Intervention | 36 | 16 | 3 | 11 |
| 9 | Control | 19 | 1 | 3 | 9 |
| 9 | Intervention | 21 | 4 | 6 | 5 |
| 12 | Control | 4 | 0 | 3 | 12 |
| 12 | Intervention | 8 | 2 | 2 | 9 |
| Totals |  | 211 | 95 | 24 | 80 |

All data are number of couples in the study at each time point, with reasons for ending study.

Covid-19: coronavirus disease 2019

**Supplementary Table S2.** Originally planned primary analysis: cumulative ongoing pregnancy within 12 months after randomisation in the subset of couples who completed our trial as planned (n=51).

| **12 months ongoing pregnancy rate in couples entering on or before March 13, 2019.** | | |
| --- | --- | --- |
|  | Control (n=26) | Intervention (n=25) |
| n (%) No ongoing pregnancy | 11 (42%) | 12 (48%) |
| n (%) Ongoing pregnancy | 15 (58%) | 13 (52%) |

| **Logistic regression of cumulative ongoing pregnancy in couples entering on or before March 13, 2019.** | | | |
| --- | --- | --- | --- |
| Characteristic | OR^1^ | 95% CI^1^ | p-value |
| Womens’ age (years) | 0.87 | 0.73, 1.01 | 0.070 |
| Womens’ BMI (kg/m²) | 0.85 | 0.72, 0.98 | 0.031 |
| Study arm (2 vs 1) | **0.93** | **0.27, 3.20** | 0.90 |
| ^1^ OR = Odds Ratio, n = Number of patients, Arm 1 = Control, Arm 2 = Intervention | | | |

**Supplementary Table S3.** Results of the mixed models on lifestyle outcomes.

| **Women** | | | | **Men** | | | |
| --- | --- | --- | --- | --- | --- | --- | --- |
| **Mixed model for overall diet quality**  (118 observations in 89 women) | | | | (88 observations in 69 men) | | | |
|  | Beta | 95% CI | p |  | Beta | 95% CI | p |
| Baseline diet quality (%) | 0.8 | 0.6, 1 | 0 | Baseline diet quality (%) | 0.9 | 0.6, 1.2 | 0 |
| Time (months) | 14 | 1.6, 26.4 | 0.035 | Time (months) | 5 | -8.7, 18.8 | 0.484 |
| Study arm (2 vs 1) | 6.4 | 2.1, 10.7 | 0.005 | Study arm (2 vs 1) | -2.8 | -9.9, 4.3 | 0.454 |
| Interaction baseline*time | -0.1 | -0.2, 0.1 | 0.305 | Interaction baseline*time | -0.2 | -0.4, 0 | 0.094 |
| Interaction arm*time | -4.1 | -7, -1.2 | 0.009 | Interaction arm*time | 4.2 | -0.3, 8.7 | 0.087 |
| **Mixed model for fruit intake**  (121 observations in 91 women) | | | | (89 observations in 70 men) | | | |
|  | Beta | 95% CI | p |  | Beta | 95% CI | p |
| Baseline fruit intake (g/day) | 0.7 | 0.4, 1.1 | 0 | Baseline fruit intake (g/day) | 0.3 | 0, 0.7 | 0.083 |
| Time (months) | 146.3 | 47.1, 245.5 | 0.006 | Time (months) | -98.2 | -195.3, -1 | 0.06 |
| Study arm (2 vs 1) | 93.7 | 25.3, 162 | 0.01 | Study arm (2 vs 1) | -62.8 | -140.6, 14.9 | 0.127 |
| Interaction baseline*time | -0.1 | -0.4, 0.2 | 0.472 | Interaction baseline*time | 0.2 | -0.1, 0.4 | 0.248 |
| Interaction arm*time | -77.6 | -128.5, -26.6 | 0.005 | Interaction arm*time | 61.4 | 3.5, 119.3 | 0.05 |
| **Mixed model for vegetable intake**  (121 observations in 91 women) | | | | (89 observations in 70 men) | | | |
|  | Beta | 95% CI | p |  | Beta | 95% CI | p |
| Baseline vegetable intake (g/day) | 0.3 | -0.1, 0.8 | 0.161 | Baseline vegetable intake (g/day) | 0.4 | 0, 0.8 | 0.055 |
| Time (months) | 41.2 | -60.4, 142.9 | 0.437 | Time (months) | 54.5 | -40.1, 149 | 0.282 |
| Study arm (2 vs 1) | 94.4 | 19, 169.8 | 0.018 | Study arm (2 vs 1) | 70 | 3.2, 136.9 | 0.049 |
| Interaction baseline*time | 0.1 | -0.2, 0.4 | 0.452 | Interaction baseline*time | 0.1 | -0.2, 0.3 | 0.66 |
| Interaction arm*time | -41.2 | -96.9, 14.6 | 0.161 | Interaction arm*time | -34.1 | -79.1, 10.8 | 0.158 |
| **Mixed model for total moderate to vigorous physical** activity (121 observations in 91 women) | | | | (89 observations in 70 men) | | | |
|  | Beta | 95% CI | p |  | Beta | 95% CI | p |
| Baseline TMVPA (min/week) | 0.4 | 0, 0.8 | 0.059 | Baseline TMVPA (min/week) | 0.3 | -0.1, 0.7 | 0.121 |
| Time (months) | 36.4 | -582.2, 654.9 | 0.91 | Time (months) | -301.9 | -1020.9, 417 | 0.429 |
| Study arm (2 vs 1) | 168.9 | -319.6, 657.4 | 0.505 | Study arm (2 vs 1) | -337.1 | -884.4, 210.3 | 0.243 |
| Interaction baseline*time | 0 | -0.2, 0.3 | 0.766 | Interaction baseline*time | 0 | -0.3, 0.3 | 0.952 |
| Interaction arm*time | -97.6 | -459.7, 264.4 | 0.606 | Interaction arm*time | 175.8 | -210.5, 562.1 | 0.392 |
| **Mixed model for sedentary behaviour**  (121 observations in 91 women) | | | | (89 observations in 70 men) | | | |
|  | Beta | 95% CI | p |  | Beta | 95% CI | p |
| Baseline sedentary behaviour (min/day) | 0.3 | -0.1, 0.8 | 0.156 | Baseline sedentary behaviour (min/day) | 0.2 | -0.2, 0.6 | 0.324 |
| Time (months) | 117.7 | -183.1, 418.5 | 0.455 | Time (months) | 27.2 | -207.2, 261.6 | 0.827 |
| Study arm (2 vs 1) | 74.9 | -119.9, 269.7 | 0.459 | Study arm (2 vs 1) | 79.8 | -104, 263.5 | 0.409 |
| Interaction baseline*time | -0.1 | -0.4, 0.3 | 0.714 | Interaction baseline*time | 0.1 | -0.2, 0.4 | 0.518 |
| Interaction arm*time | -66.1 | -209.6, 77.5 | 0.379 | Interaction arm*time | -29.6 | -162, 102.8 | 0.673 |
| **Mixed model for emotional distress**  (120 observations in 90 women) | | | | (89 observations in 70 men) | | | |
|  | Beta | 95% CI | p |  | Beta | 95% CI | p |
| Baseline Total DASS-21 score | 0.5 | 0.2, 0.8 | 0.001 | Baseline Total DASS-21 score | 0.7 | 0.2, 1.2 | 0.012 |
| Time (months) | -3.9 | -17.8, 9.9 | 0.585 | Time (months) | -7.2 | -24.8, 10.4 | 0.439 |
| Study arm (2 vs 1) | -5.7 | -16.4, 5 | 0.303 | Study arm (2 vs 1) | -7.9 | -22, 6.2 | 0.288 |
| Interaction baseline*time | 0.2 | 0, 0.5 | 0.063 | Interaction baseline*time | -0.1 | -0.5, 0.3 | 0.504 |
| Interaction arm*time | 2.8 | -4.7, 10.3 | 0.479 | Interaction arm*time | 6.2 | -4.2, 16.7 | 0.26 |
| **Mixed model for fertility related quality of life**  (120 observations in 90 women) | | | | (89 observations in 70 men) | | | |
|  | Beta | 95% CI | p |  | Beta | 95% CI | p |
| Baseline Total FERTIQOL score | 0.7 | 0.5, 1 | 0 | Baseline Total FERTIQOL score | 0.9 | 0.5, 1.3 | 0 |
| Time (months) | -1.6 | -16.8, 13.6 | 0.839 | Time (months) | 14.3 | -14.8, 43.5 | 0.356 |
| Study arm (2 vs 1) | 2.8 | -3.9, 9.4 | 0.417 | Study arm (2 vs 1) | 7.4 | -0.8, 15.6 | 0.09 |
| Interaction baseline*time | 0 | -0.2, 0.2 | 0.961 | Interaction baseline*time | -0.1 | -0.4, 0.3 | 0.691 |
| Interaction arm*time | -1.3 | -5.9, 3.3 | 0.593 | Interaction arm*time | -6.5 | -12.6, -0.5 | 0.043 |
| **Mixed model for BMI**  (216 observations in 170 women) | | | | (205 observations in 162 men) | | | |
|  | Beta | 95% CI | p |  | Beta | 95% CI | p |
| Baseline BMI (kg/m²) | 1 | 0.9, 1 | 0 | Baseline BMI (kg/m²) | 1 | 0.9, 1.1 | 0 |
| Time (months) | 0.1 | -0.2, 0.4 | 0.515 | Time (months) | 0.3 | -0.5, 1.1 | 0.434 |
| Study arm (2 vs 1) | 0.1 | -0.3, 0.4 | 0.723 | Study arm (2 vs 1) | 0.3 | -0.3, 0.8 | 0.335 |
| Interaction baseline*time | 0 | 0, 0 | 0.706 | Interaction baseline*time | 0 | 0, 0 | 0.795 |
| Interaction arm*time | -0.1 | -0.2, 0 | 0.17 | Interaction arm*time | -0.1 | -0.4, 0.1 | 0.194 |
| **Mixed model for waist circumference**  (213 observations in 168 women) | | | | (213 observations in 168 men) | | | |
|  | Beta | 95% CI | p |  | Beta | 95% CI | p |
| Baseline waist circumference (cm) | 0.8 | 0.7, 0.9 | 0 | Baseline waist circumference (cm) | 0.8 | 0.7, 0.9 | 0 |
| Time (months) | 1.9 | -2.2, 5.9 | 0.37 | Time (months) | -1.6 | -5.3, 2 | 0.386 |
| Study arm (2 vs 1) | 1 | -1.7, 3.8 | 0.462 | Study arm (2 vs 1) | 2.5 | 0.4, 4.5 | 0.022 |
| Interaction baseline*time | 0 | -0.1, 0 | 0.56 | Interaction baseline*time | 0 | 0, 0.1 | 0.215 |
| Interaction arm*time | -0.4 | -1.5, 0.7 | 0.513 | Interaction arm*time | -0.3 | -1.2, 0.5 | 0.429 |
| Arm 1 = Control, Arm 2 = Intervention; , DASS = Depression, Anxiety and Stress Scale, n = Number of patients | | | | | | | |

.

**Supplementary Table S4.** Actual use of the mobile application part of the PreLiFe-programme.

| **Use** | **Women**  **n (%)** |  | **Men**  **n (%)** | |  | **Couple**  **n (%)** |  |
| --- | --- | --- | --- | --- | --- | --- | --- |
| n (%) Food module used (receiving tips and at least 1 goalsetting on food literacy) | 79 | (75%) | 38 | (36%) | | 31 | (29%) |
| n (%) Physical activity (PA) module used (receiving tips and at least 1 registration of step count or monitoring of PA) | 75 | (71%) | 50 | (47%) | | 37 | (35%) |
| n (%) Mindfulness module used (receiving tips and at least 1 exercise followed) | 70 | (66%) | 39 | (37%) | | 32 | (30%) |
| **n (%) that actively used all modules** | **52** | (49%) | **20** | (19%) | | **15** | (14%) |
| **n (%) that did not actively use any module** | **10** | (9%) | **40** | (38%) | | **5** | (5%) |


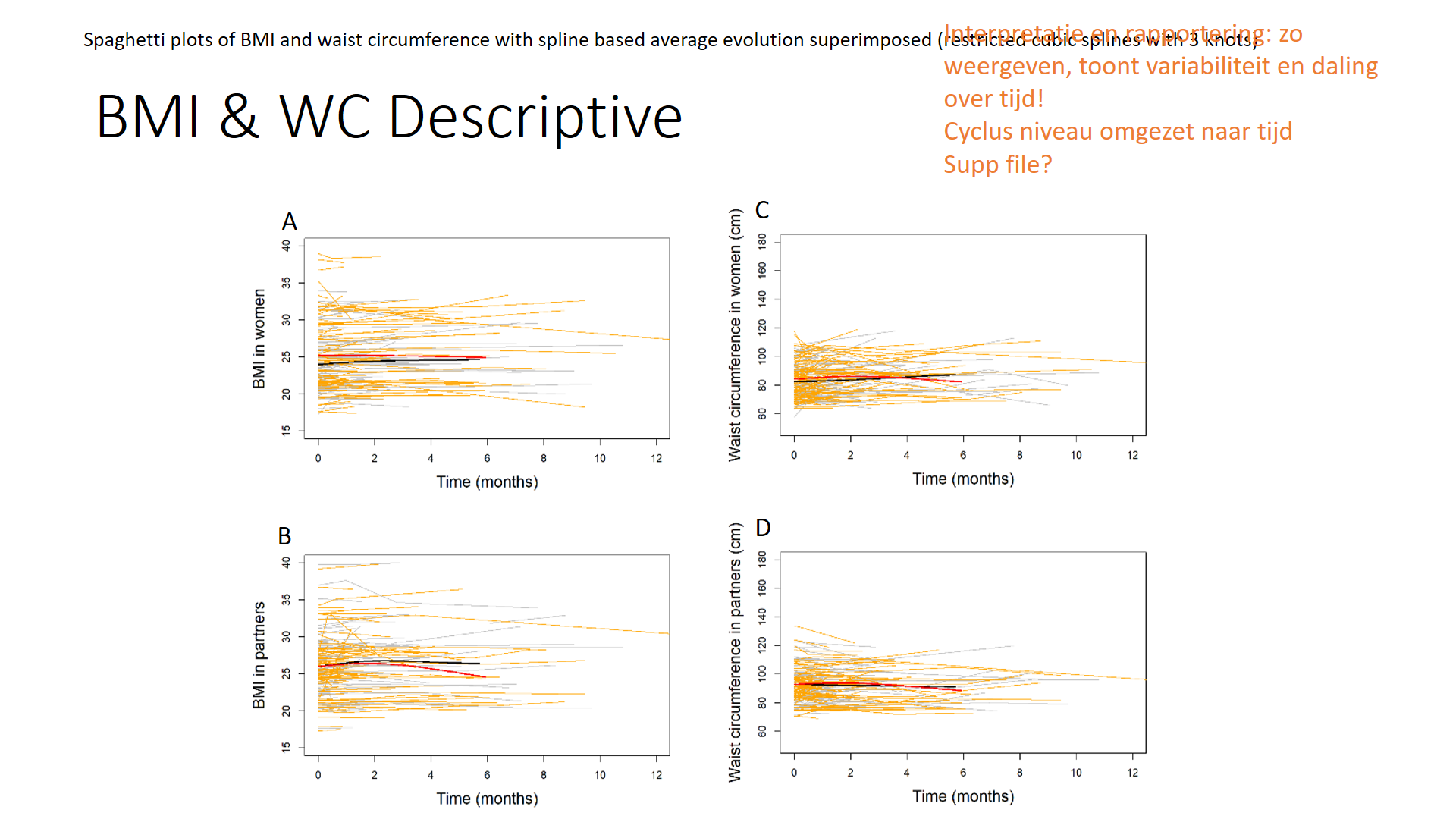


**Supplementary Figure S1.** Descriptive representation of BMI and waist circumference.

Spaghetti plots of BMI and waist circumference with spline-based average evolution superimposed (restricted cubic splines with 3 knots). **A.** BMI in women, **B**. BMI in men**, C.** Waist circumference in women, **D.** Waist circumference in men

Time refers to time from randomisation.
